# Angulation of the dural venous sinuses in the posterior cranial fossa: an anatomical study and its implications for venous circulation

**DOI:** 10.1007/s10143-025-03195-9

**Published:** 2025-01-21

**Authors:** Juan J. Cardona, Rarinthorn Samrid, Chung Yoh Kim, Yoko Tabira, Aaron S. Dumont, Joe Iwanaga, R. Shane Tubbs

**Affiliations:** 1 Department of Neurosurgery, Clinical Neuroscience Research Center, Tulane University School of Medicine, New Orleans, LA USA; 2 Division of Gross and Clinical Anatomy, Department of Anatomy, Kurume University School of Medicine, Kurume, Fukuoka Japan; 3 Department of Neurology, Clinical Neuroscience Research Center, Tulane University School of Medicine, New Orleans, LA USA; 4 Department of Structural & Cellular Biology, Tulane University School of Medicine, New Orleans, LA USA; 5 Department of Neurosurgery and Ochsner Neuroscience Institute, Ochsner Health System, New Orleans, LA USA; 6 Dental and Oral Medical Center, Kurume University School of Medicine, Kurume, Fukuoka Japan; 7 Department of Anatomical Sciences, St. George’s University, St. George’s, Grenada; 8 Department of Surgery, Tulane University School of Medicine, New Orleans, LA USA; 9 University of Queensland, Brisbane, Australia; 10 Department of Anatomy, Faculty of Medicine, Khon Kaen University, Khon Kaen, Thailand; 11 Department of Anatomy, Dongguk University School of Medicine, Gyeongju, Republic of Korea

**Keywords:** Angulation, Transverse sinus, Idiopathic intracranial hypertension, Sinus stenosis, Sinus thrombosis, Pulsatile tinnitus

## Abstract

The purpose of the current study was to determine the angulation of the dural venous sinuses in soft tissue, to evaluate differences between types of tissue, and to discuss the potential influence of these angulations on intracranial venous hemodynamics and related pathologies. Angulations formed in different segments of the transverse, sigmoid, and superior sagittal sinuses were measured in 13 adult human cadaveric heads (26 sides). After the soft tissues were removed, measurements were also taken from the underlying bone. The overall angulation of the transverse sinus was assessed using two reference points, while the lengths and widths of the dural venous sinuses were measured using microcalipers. Statistical analyses were performed considereing sides, sex, and types of tissue. The mean angulation of the superior sagittal sinuses - transverse sinus junction was 116 degrees. The mean angulations of the transverse sinus - sigmoid sinus junction in medial and superior views were 108 degrees and 114 degrees, respectively. The mean angulations of the entire transverse sinus at two different points were 45 degrees and 44 degrees, respectively. There were statistically significant differences in angulation in some variables when they were adjusted for sides and sex, but not types of tissue. Angulation is a paramount factor in venous hemodynamics. Certain angulations of the dural venous sinuses differed significantly between sides and sexes, but not between types of tissue. Future research should investigate the effects of these angulations on intracranial venous circulation and their relevance to related pathologies.

## Introduction

Stroke is the second-leading cause of death (11.6%; [10.8–12.2] of total deaths), after ischemic heart disease, and the third-leading cause of disability and death combined worldwide [1]. Its counterpart, cerebral vein and dural venous sinus thrombosis (CVST), is an underdiagnosed and less common cause of stroke (0.5-1% of all strokes). The incidence of this venous disorder has been increasing during the last few years owing to enhancements in its diagnostic approach and emergent cases secondary to either a post-SARS-CoV-2 vaccine reaction or a complication of the respiratory infection itself [2, 3].

Several studies have identified prothrombotic risk factors for CVST such as cancer (OR 4.86; 95% CI 3.46–6.81), oral contraceptive use (OR 5.59; 3.95–7.91), Factor V Leiden mutation (OR 3.38; 2.27–5.05), mutation of G20210A (OR 9.27; 5.85–14.67), hyperhomocysteinemia (OR 4.07; 2.54–6.52), antithrombin deficiency (OR 3.8; 1.0-13.8), protein C (OR 10.7; 3.1–37.7) and S (OR 5.7; 1.4–22.4) deficiency, etc. In contrast, CVST is reported to be idiopathic in up to 44% of cases [4, 5–8, 9, 10]. Overall, its etiology is considered multifactorial, and each factor causes or triggers thrombosis through complex and only partially-understood mechanisms. Furthermore, some studies have described anatomical factors (intraluminal or intrinsic, and extraluminal or extrinsic) potentially related to CVST and other dural venous sinus (DVS) pathologies; these factors mostly involve the transverse sinus (TS), including hypoplastic-absent TS, venous sinus stenosis (VSS), arachnoid granulations, trabeculae/septa, blind pouches, bony prominence, etc [11, 12, 13].

In light of this, we proposed angulation of the DVSs in the posterior cranial fossa as another intrinsic anatomical factor and pioneered the measurement of this parameter in the grooves of skulls = bone tissue [14]. In general, it has been hypothesized that these structural abnormalities, including angulation, are likely to interfere with the hemodynamics of DVSs, thus conditioning the internal walls of venous sinuses to endothelial changes, VSS, venous stasis, and finally a higher risk of thrombosis and the development of other DVS blood flow-associated conditions such as idiopathic intracranial hypertension (IIH), venous sinus diverticulum (VSD), and pulsatile tinnitus (PT) [11, 14, 12, 13]. Therefore, since we have already measured angulations in bone tissue but could not explore the correlation with soft tissue, the aims of the present study were (1) to investigate angulation in multiple segments of the DVSs in the posterior cranial fossa in both soft tissue and the underlying bony grooves, (2) to compare the findings in these two tissues, and (3) to discuss the potential role of this factor in the hemodynamics of DVSs and associated pathologies.

## Materials and methods

### Specimens and dissection

Thirteen adult human heads (eight females and five males; *n* = 26 sides) derived from ten formalin-fixed and three fresh-frozen cadavers were used for this study; four of them were latex-injected. The mean age at death was 83 years (range 57–98 years). The medical history of the cadavers was not available. Traumatic stigmata were not identified on the heads, and the posterior cranial fossa was not dissected in any specimen. Each specimen underwent a circumferential craniotomy above the pinnae, using an electric autopsy saw (Stryker Inc., Kalamazoo, MI, USA) to excise the cranial bone overlying the supratentorial neural structures. Subsequently, the neural tissue was dissected to reveal the DVSs within the posterior cranial fossa. The DVSs with any damage due to previous dissection or pathology were excluded from this study. The DVSs were then meticulously transected in their entirety to expose their internal structural features, and the latex in the four latex-injected specimens was examined.

### Angulations

The methods used in our previous study of angulations in the DVSs grooves were applied to measure all the parameters herein [14]. In brief: angulations were obtained using photographic images taken in four different planes (plane 1: parallel to the confluence of the sinuses; plane 2: parallel to the posterior cranial fossa; and planes 3 and 4: parallel to the right and left transverse-sigmoid sinus junction (TS-SSJ), respectively). The total number of angles measured in the present study was 18:

- Plane 1: Superior sagital-transverse sinus junction (SSS-TSJ), bilaterally (two angles).
- Plane 2: TS1, TS2, TS3, TS-SSJsv, SS, TSh, and TSv, bilaterally (14 angles).
- Plane 3: TS-SSJmv right side (one angle).
- Plane 4: TS-SSJmv left side (one angle).

### Other measurements and statistical analyses

All the parameters were measured again in the underlying bone (DVS grooves; 26 sides) after the soft tissue was removed, in order to compare the findings between the two tissues. Additionally, the lengths and widths of the DVSs in the same segments of angulation were measured using microcalipers (Mitutoyo, Japan). Two authors obtained the measurements meticulously, three times each. R Statistical Software (v4.4.0; R Core Team 2024) for macOS was used for statistical analyses (Chi-square test), statistical significance being set at *p* < 0.05 for sides, sexes, and types of tissue. The general results reported in the first part below are limited to soft tissue. In contrast, the statistical analyses compared the two groups. The authors state that every effort was made to follow all local and international ethical guidelines and laws that pertain to the use of human cadaveric donors in anatomical research [15].

## Results

**Fig. 1:**
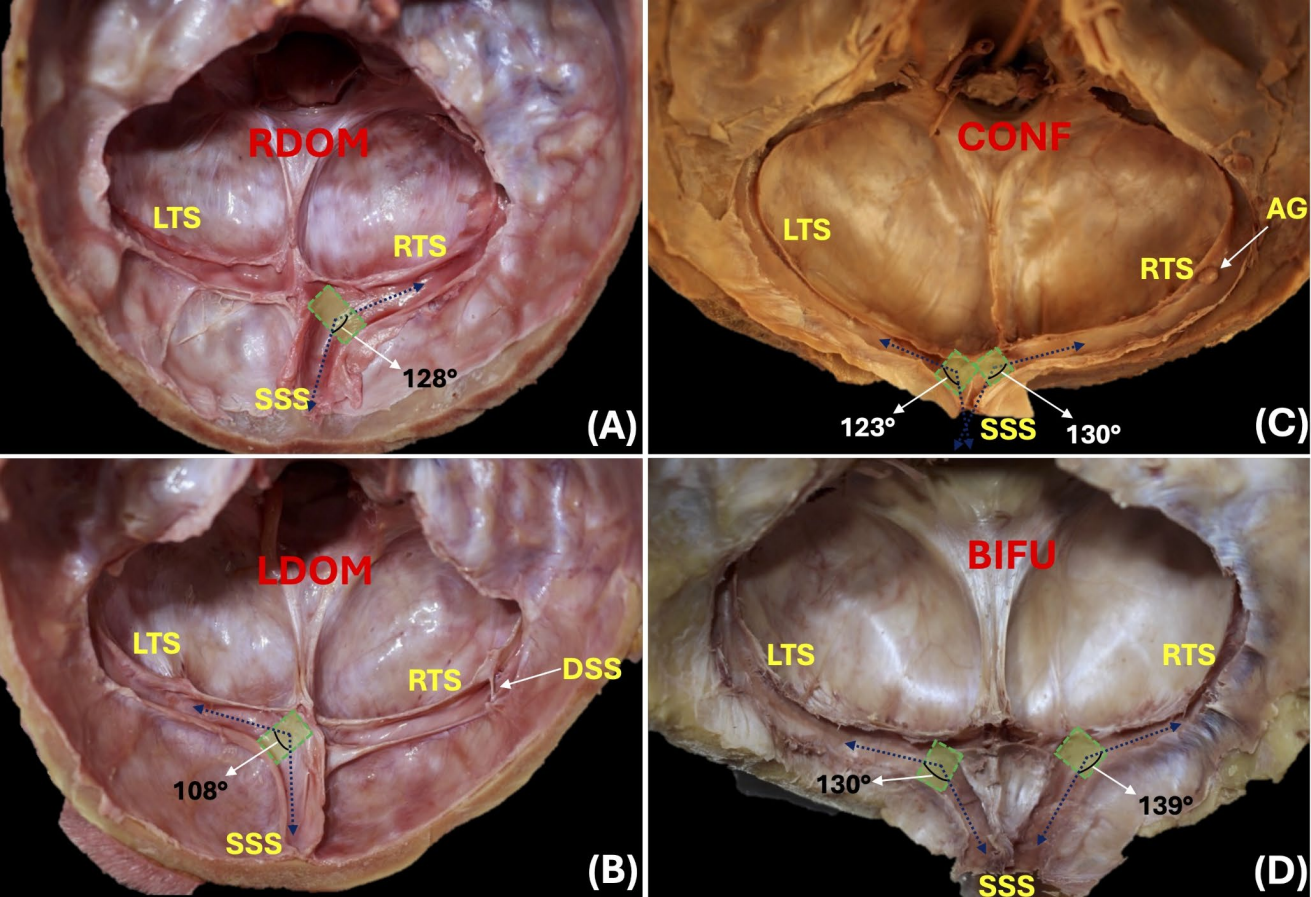
Plane 1. Parallel to the confluence of the sinuses. Note the area of the junction between the SSS and TS (green square), and the angulation of the SSS-TSJ measured in four skulls with right-dominant (RDOM; 1**A**), left-dominant (LDOM; 1**B**), confluence (CONF; 1**C**), and bifurcation (BIFU; 1D) patterns of communication. Also, note the presence of a huge arachnoid granulation (AG; 1**C**) and a type I-class I dural sinus septum (DSS; 1**B**). LTS: Left transverse sinus; RTS: Right transverse sinus; SSS: Superior sagittal sinus

**Fig. 2:**
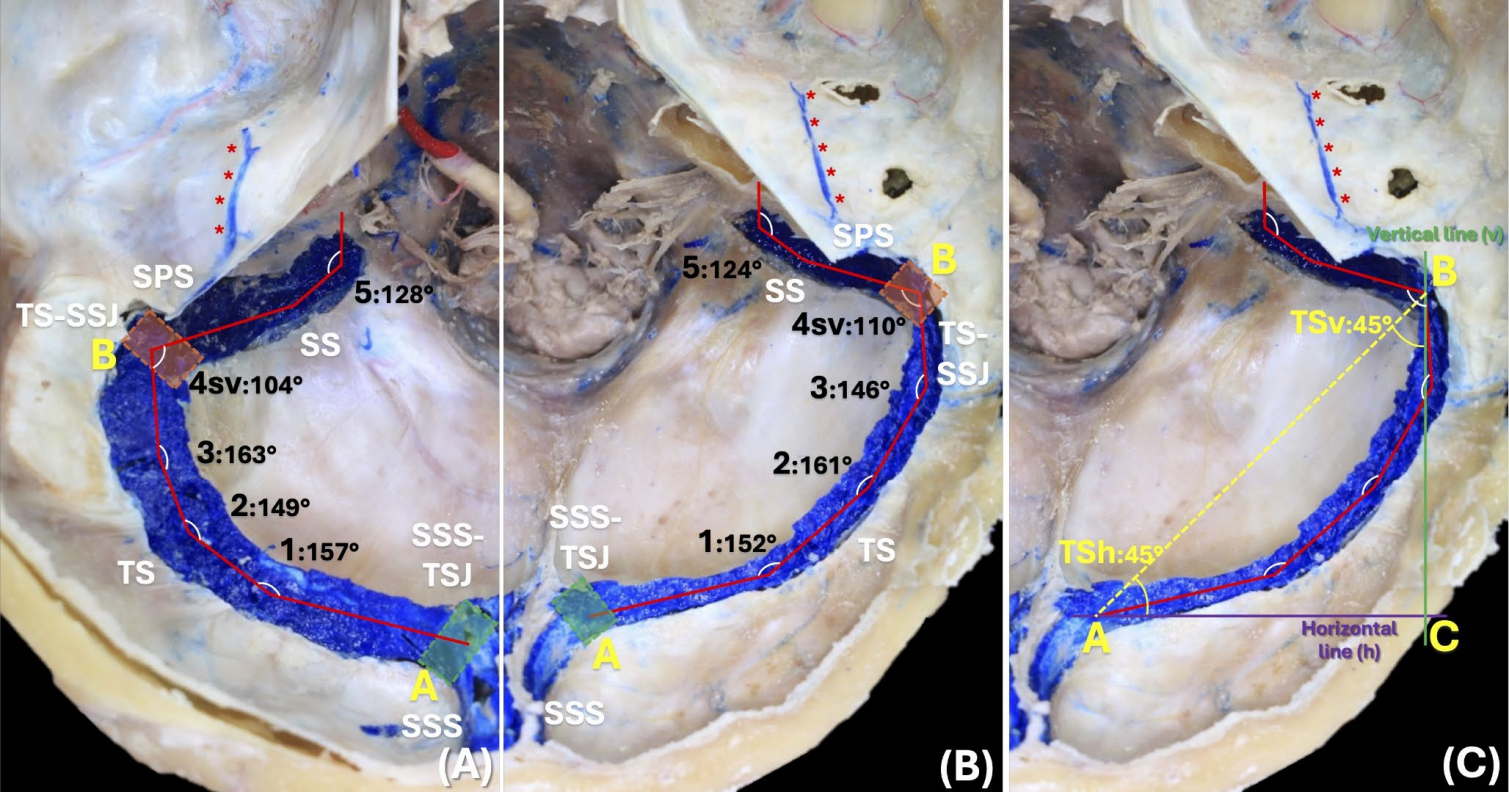
Plane 2. Parallel to the posterior cranial fossa. Point A is located at the beginning of the proximal third of the TS, or the SSS-TS junction (SSS-TSJ; green square), and point B is located at the TS-SS junction (TS-SSJ; orange square). 2**A** and 2B: Measurement of angulations at points 1 (TS1 angle), 2 (TS2 angle), 3 (TS3 angle), 4sv (TS-SSJsv angle), 5 (SS angle) on the left (2**A**) and right (2**B**) sides. 2**C**: Drawing of two imaginary lines: vertical (green; v) and horizontal (purple; h) intersecting points B and A, respectively. A straight line was also drawn joining points A and B (yellow dashed line), and the entire angulations of the right TS at point A (TSh angle) formed by BAC and at point B (TSv angle) formed by ABC were measured. Note the bilateral venous sinuses of Kelch (red asterisks). TS; Transverse sinus; SS: Sigmoid sinus; SSS: Superior sagittal sinus; SPS: Superior petrosal sinus

**Fig. 3:**
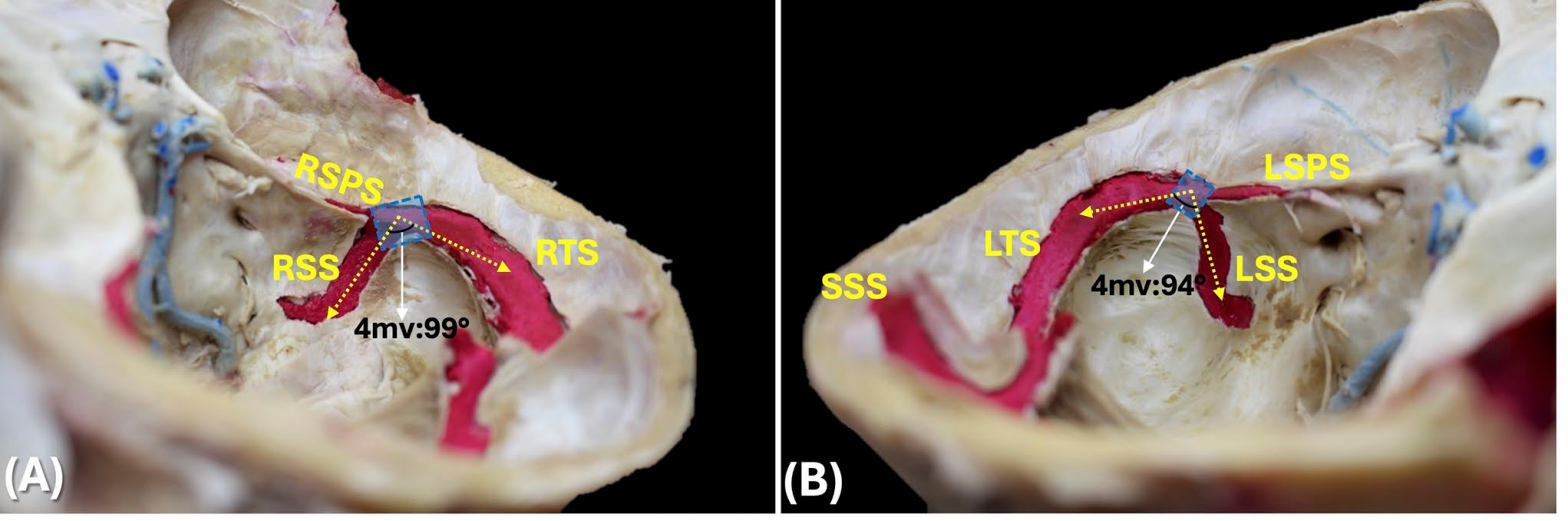
Planes 3 and 4: Parallel to the right and left TS-SSJ, respectively. Note the area of the junction between the TS and SS (blue squares), and the angulation of the TS-SSJ in a medial view (TS-SSJmv angle) measured on the right (3**A**) and left (3**B**) sides. Also, note the difference in size between the left superior petrosal sinus (LSPS) and the right (RSPS). LTS: Left transverse sinus; RTS: Right transverse sinus; LSS: Left sigmoid sinus; RSS: Right sigmoid sinus

In plane 1, the most common communication pattern at the confluence of the sinuses was right-dominant (*n* = 8; 61.6%), followed by left-dominant (*n* = 2; 15.3%) and bifurcation (*n* = 2; 15.3%). Also, the mean angulation of the SSS-TSJ angle in plane 1 was 116 ± 6.21 degrees (range 107–132 degrees) (Fig. 1). In plane 2, the mean angulations of the TS1, TS2, and TS3 angles were 155 ± 7.35 degrees (range 140–180 degrees), 152 ± 6.24 degrees (range 144–168 degrees), and 146 ± 5.38 degrees (range 137–156 degrees), respectively. Those of the TS-SSJsv and SS angles were 114 ± 11.58 degrees (range 96–131 degrees) and 123 ± 7.03 degrees (range 113–138 degrees), respectively. Also, in plane 2 the mean angulations of the whole TS represented by the TSh and TSv angles were 45 ± 4.74 degrees (range 39–64 degrees) and 44 ± 4.74 degrees (range 26–51 degrees), respectively (Fig. 2). In planes 3 and 4, the mean angulation of the TS-SSJmv angle was 108 ± 11.65 degrees (range 90–124 degrees) (Fig. 3).

The mean lengths of the TS and SS were 61.55 ± 2.78 millimeters (mm) (range 57.32–67.88 mm) and 34.13 ± 1.90 mm (range 31.06–36.85 mm), respectively. The mean widths at the TS-SSJ and SSS-TSJ were 8.78 ± 1.92 mm (range 5.98–13.62 mm) and 8.55 ± 1.39 mm (range 5.82–11.43 mm), respectively. Other morphometric measurements and the overall results of angulation limited to soft tissue are presented in Table 1.

**Table 1:** Overall angulation and morphometric data of the dural venous sinuses of the posterior cranial fossa (limited to soft tissue)

Angle	Mean (SD) degrees	Median (IQR) degrees	Range degrees
TS1 (*n* = 26)	155 (7.35)	154 (151–156)	140–180
TS2 (*n* = 26)	152 (6.24)	153 (146–155)	144–168
TS3 (*n* = 26)	146 (5.38)	146 (144–150)	137–156
TS-SSJsv (*n* = 26)	114 (11.58)	111 (105–128)	96–131
TS-SSJmv (*n* = 26)	108 (11.65)	107 (97–118)	90–124
SS (*n* = 26)	123 (7.03)	123 (118–128)	113–138
SSS-TSJ (*n* = 16)	116 (6.21)	118 (112–119)	107–132
TSh (*n* = 26)	45 (4.74)	45 (43–47)	39–64
TSv (*n* = 26)	44 (4.74)	45 (43–46)	26–51
**Width (W)/ Length (L)**	**Mean (SD) mm**	**Median (IQR) mm**	**Range mm**
W TS1 (*n* = 26)	5.57 (1.98)	5.5 (3.8–7.4)	2.79–8.45
W TS2 (*n* = 26)	6.20 (1.69)	5.7 (4.7–7.2)	4.28–11.25
W TS3 (*n* = 26)	7.62 (1.58)	7.4 (6.2–8.3)	5.93–11.94
W TS-SSJ (*n* = 26)	8.78 (1.92)	8.5 (7.2–9.6)	5.98–13.62
W SSS-TSJ (*n* = 16)	8.55 (1.39)	8.1 (8.1–9.2)	5.82–11.43
W pro SS (*n* = 26)	10.13 (1.55)	10.1 (8.8–11.0)	7.36–13.35
W distal SS (*n* = 26)	6.54 (1.95)	6.1 (5.7–7.7)	3.89–11.01
L SS (*n* = 26)	34.13 (1.90)	34.3 (33.3–35.8)	31.06–36.85
L TS (*n* = 26)	61.55 (2.78)	61.3 (59.5–61.9)	57.32–67.88

TS: transverse sinus, SS: sigmoid sinus, SSS: superior sagittal sinus, SSJ; sigmoid sinus junction, TSJ: transverse sinus junction, sv: superior view, mv: medial view

### Statistical analyses (sides, sexes, and types of tissue)

**Fig. 4:**
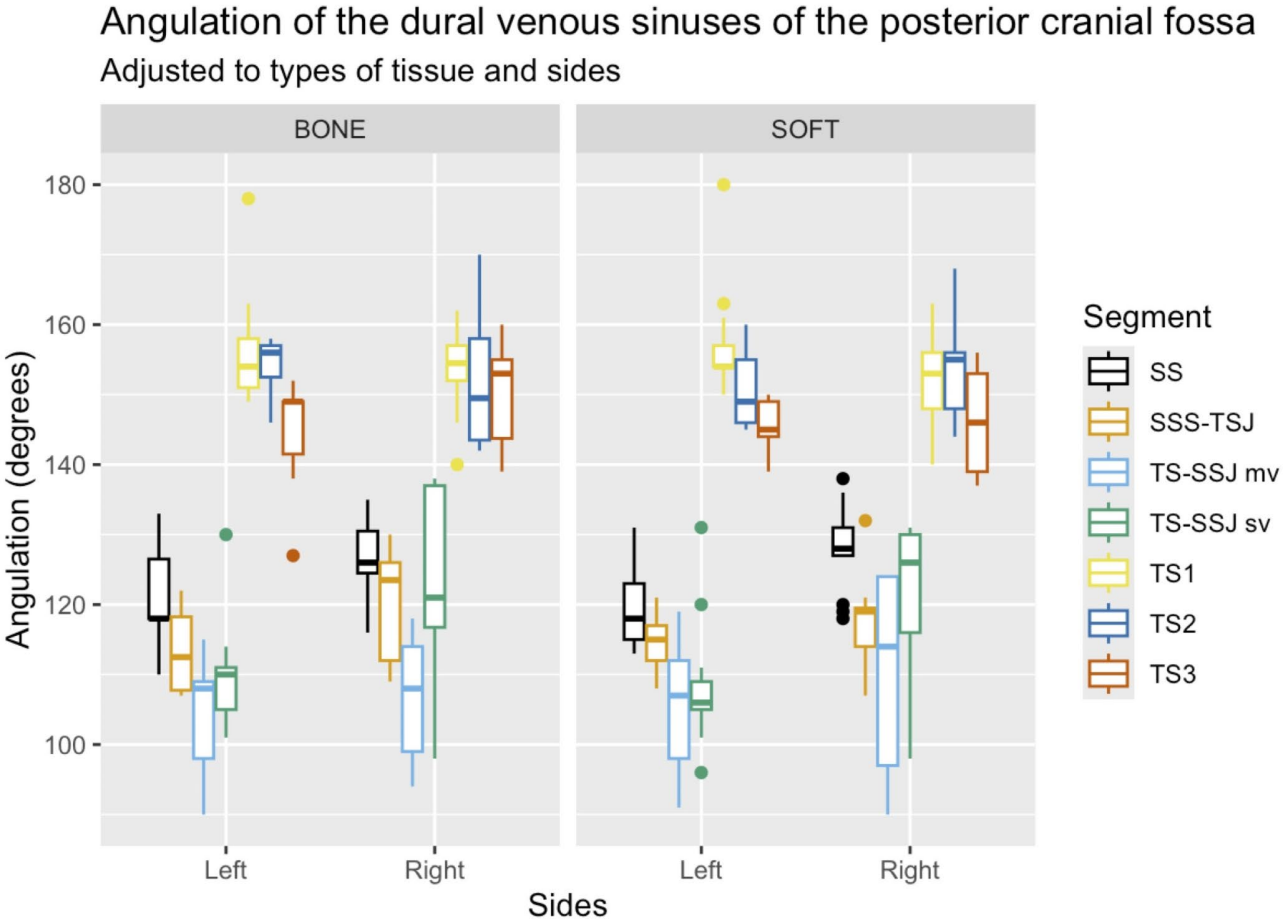
Boxplot depicting the angulation of the dural venous sinuses of the posterior cranial fossa adjusted to types of tissue and sides

Some angulations differed significantly between sides and sexes: the mean angulations of the TS-SSJsv angle on the left and right sides were 109 ± 8.17 degrees (range 96–131 degrees) and 122 ± 11.11 degrees (range 99–138 degrees), respectively; in addition, this segment showed significant differences in the medial view (TS-SSJmv) and when it was adjusted for sex. Similarly, the SS angle appeared remarkably different in both comparisons (sides and sexes). Moreover, the overall angulations of the TS in females and males were: TSh angle: 44 ± 2.81 degrees (range 39–49 degrees) and 47 ± 6.29 degrees (range 40–64 degrees), respectively; TSv angle: 45 ± 2.81 degrees (range 41–51 degrees) and 42 ± 6.29 degrees (range 26–50 degrees), respectively. Some morphometric data, such as the width at TS1 and the lengths of the SS and TS, differed notably between sides. Lastly, the statistical analysis between types of tissue showed significant differences only in the widths at points TS2 and TS3. Thus, any of the angulations in different segments were statistically significant (Fig. 4). All the statistically significant results (angles and morphometric data) obtained in this study are summarized in Table 2.

**Table 2:** Statistically significant results in angulation and morphometric data for the dural venous sinuses of the posterior cranial fossa

Angle𝛺,𝞍,&	Mean (SD) degrees		Median (IQR) degrees		Range degrees		*P* value
(*n*=♀,<,𝛽 / ♂,>,𝛿)	♀,<,𝛽	♂,>,𝛿	♀,<,𝛽	♂,>,𝛿	♀,<,𝛽	♂,>,𝛿	
TS-SSJsv𝝓 (49 = 24/25)	109 (8.17)	122 (11.11)	108 (105–111)	122 (116–131)	96–131	99–138	0.0000
TS-SSJmv𝝓 (49 = 24/25)	106 (9.71)	117 (8.21)	107 (97–111)	114 (99–118)	90–119	90–124	0.0421
TS-SSJmv& (49 = 29/20)	102 (10.27)	113 (5.99)	99 (95–108)	114 (109–118)	90–124	99–124	0.0001
SS𝝓 (49 = 24/25)	120 (5.99)	127 (6.03)	118 (115–125)	128 (126–131)	110–133	116–138	0.0003
SS& (49 = 29/20)	125 (6.31)	120 (6.79)	126 (120–130)	118.5 (115–127)	115–138	110–135	0.0198
TSh& (49 = 29/20)	44 (2.81)	47 (6.29)	44 (43–46)	47 (44–48)	39–49	40–64	0.0461
TSv& (49 = 29/20)	45 (2.81)	42 (6.29)	46 (44–47)	42.5 (42–46)	41–51	26–50	0.0423
**Width (W)/ Length (L) (n=**♀,<,𝛽 / ♂,>,𝛿)	**Mean (SD) mm**		**Median (IQR) mm**		**Range mm**		***P*** **value**
	♀,<,𝛽	♂,>,𝛿	♀,<,𝛽	♂,>,𝛿	♀,<,𝛽	♂,>,𝛿	
W TS2𝛺 (47 = 21/26)	7.26 (1.82)	6.19 (2.85)	6.72 (6.67–8.11)	5.7 (4.74–7.32)	4.50-11.82	4.28–11.20	0.0461
W TS3𝛺 (47 = 21/26)	8.56 (1.42)	7.62 (2.48)	8.20 (8.04–8.61)	7.44 (6.28–8.26)	6.55–12.51	5.93–11.90	0.0353
W TS1𝝓 (48 = 24/24)	5.19 (1.98)	6.51 (1.72)	4.44 (3.88–7.32)	6.85 (4.87–7.55)	2.79–8.55	3.24–9.67	0.0178
L SS𝝓 (49 = 24/25)	33.70 (2.29)	35.19 (1.53)	33.40 (31.43–35.79)	33.55 (34.35–35.87)	31.06–37.23	33.29–39.27	0.0114
L TS𝝓 (49 = 24/25)	63.74 (2.49)	60.49 (2.08)	64.82 (61.34–66.40)	59.57 (58.99–61.96)	60.12–67.88	57.32-66.00	0.0000

𝝓: Statistical analysis based on sides= <: Left side / >: Right side

&: Statistical analysis based on sex= ♀: Female / ♂: Male

𝛺: Statistical analysis based on types of tissue= 𝛽: Bone tissue / 𝛿: Soft tissue

TS: transverse sinus, SS: sigmoid sinus, SSS: superior sagittal sinus, SSJ; sigmoid sinus junction, TSJ: transverse sinus junction, sv: superior view, mv: medial view

## Discussion

The DVSs are crucial for maintaining the balance of venous blood within the skull by facilitating venous drainage through the lateral sinuses (TS-SS) as they empty into the internal jugular vein via the jugular foramen. Understanding the normal blood flow, its distinct features, and its potential variations is essential for comprehending how DVS blood behaves intraluminally. Moreover, such knowledge could help to establish potential connections between theoretical concepts and practical observations in the context of pathologies associated with DVS blood flow. The study by Okudera et al. reported that during the 17th -20th week of fetal development, the enlargement of the lateral border of the transverse sinus on both sides can be significant, extending posteriorly towards the superior sagittal-petrosal sinus, which contributes to an increase in brain volume [16]. Abnormal development during 30th -40th week can result in variations in the venous sinuses, leading to an increased volume of the posterior cranial fossa [17]. After birth, the transition to an erect posture can affect the angle of the jugular sinus [16]. Developmentally, the total venous blood volume is derived from the sigmoid and inferior petrosal sinuses [18]. Additionally, chordae Willisii are commonly found in the superior and transverse sinuses, where they help regulate the sinus flow and assist in thrombus organization [12]. These chordae also help prevent retrograde flow within the sinuses [12]. In addition, the DVS develops through a mesh-like formation [19]. The merging tributaries from the anterior and middle dural plexuses originate in the superior and inferior sagittal sinuses, contributing to the formation of the sagittal plexus [19]. This developmental process may lead to abnormalities, and the plexiform arrangement of the sinuses can persist into adulthood [19].

Schuchardt et al. quantified physiological DVS flow in vivo in 15 volunteers [20]. They found that the venous flow velocity and volume (mean ± SD) in the left TS (0.07 ± 0.05 m/s) (0.0018 ± 0.0015 L/s) were significantly lower than the right (0.11 ± 0.05 m/s) (0.0035 ± 0.0018 L/s) (*p* < 0.01). Similarly, Mehta et al. described normal physiological variations of DVS flow [21]. They found that both deep inspiratory and expiratory breath-holding decreased the venous flow velocity and volume in the TS and SSS notably compared with regular breathing. In the TS, the venous flow velocity and volume in the right TS (13.03 ± 5.77 cm/s) (7.05 ± 2.70 ml/s) during regular breathing were greater than the left (10.77 ± 4.25 cm/s) (5.76 ± 3.14 ml/s).

Furthermore, Dai et al. recently documented the hemodynamic characteristics of the DVSs using four-dimensional flow MRI in the general population (99 healthy volunteers with specific inclusion and exclusion criteria; 55 females = 55.6%) with a mean age of 42.88 ± 13.16 years [22]. Among all their objectives and measurements, the authors employed 16 standardized planes of the DVSs to measure the blood flow velocity (V_max_, cm/s; V_avg_, cm/s) and average blood flow rate (Q, mL/s) at the SSS near the confluence, TS near the TS-SSJ, and SS and jugular bulb bilaterally. The results were as follows: SSS (16.91 ± 4.74 cm/s; 14.76 ± 4.03 cm/s)-(4.16 ± 0.97 mL/s), right TS (19.67 ± 6.06 cm/s; 17.28 ± 5.55 cm/s)-(5.33 ± 2.36 mL/s), right SS (21.38 ± 5.73; 18.37 ± 5.09 cm/s)-(4.95 ± 2.86 mL/s), right jugular bulb (18.15 ± 4.31 cm/s; 16.03 ± 4.02 cm/s)-(4.32 ± 2.30 mL/s), left TS (16.65 ± 5.45 cm/s; 14.47 ± 4.91 cm/s)-(3.65 ± 2.09 mL/s), left SS (19.20 ± 4.46 cm/s; 16.61 ± 3.84 cm/s)-(3.41 ± 1.85 mL/s), and left jugular bulb (15.70 ± 3.95 cm/s; 13.65 ± 3.86 cm/s)-(3.03 ± 1.71 mL/s). They also recorded the vortex flow pattern, defined as a completely closed ring-shaped spin loop. This parameter was measured at the confluence of the sinuses, the TS-SSJ, and the jugular bulb in 12 cases (12/99 or 12.12%; seven females and five males), 15 cases (15/169 or 8.9%; nine females and six males; nine right and six left), and 101 cases (101/169 or 59.8%; 24 left, 35 right, and 21 bilateral), respectively. Of note, the vortex flow patterns were seen at vascular junctions; however, the preponderance was low at the confluence of sinuses and at the TS-SSJ, where angulations are considerably greater than the angulation near the jugular bulb, which approximates to the SS angle (123 ± 7.03 degrees; range 90–124 degrees) obtained herein. In addition, the vortex flow patterns were identified in the normal population. Therefore, they probably also explain the low prevalence of this finding at junctions, since we hypothesize that this cohort does not present the particular intracranial venous anatomy that could potentially increase the likelihood of developing abnormal DVS blood flow patterns and DVS blood flow-related pathologies.

Importantly, these results change with the cardiac cycle even in healthy volunteers, and some of them also differ according to sex and age [22]. In the current study, we found that angulation differed significantly between females and males in some angles (TS-SSJmv and SS) and in the whole angulation of the TS (TSh and TSv). Similarly, the hemodynamics of DVSs could be altered owing to morphological changes in intracranial venous anatomy, including anatomical variations of the DVSs, patterns of communication at the confluence, and extrinsic and intrinsic intraluminal factors such as angulation [11, 14, 12, 13]. For instance, segments with faster and laminar flow could predominate for non-acute angulations, whereas segments with slow and turbulent-vortex flow could predominate for acute angulations, especially at vascular junctions. Of note, turbulent-vortex flow has been documented by Dai et al. in healthy individuals, showing a low prevalence as mentioned; in other studies it has been identified in patients with different DVS pathologies [22]. Nevertheless, the relationship with angulation has not been investigated [23, 24, 25]. In the present study, the association between angulation and DVS hemodynamics has been discussed in the foregoing, and the association with DVS blood-flow related pathology will be discussed in the following section.

### CVST, VSS, IIH, VSD, and PT

CVST is commonly reported as a single entity, the term encompassing the partial or complete obstruction of a cerebral vein and/or sinus, but it is paramount to characterize this condition further (e.g., involvement of single or multiple cerebral veins and/or DVSs, side of thrombosis, location of the affected venous structure). A systematic review by Paybast et al. included 339 patients and reported multiple-sinus and single-sinus involvement in 68.84% and 32.15% of cases, respectively [10]. Bezerra et al. described the TS as the most frequently thrombosed (356/685, 52%), followed by the SSS (400/788, 50.7%), and then the SS (251/812, 30.9%) [4]. In addition, the right TS was involved in 49.5% of cases (95/192) and the left TS in 55.7% (107/192). On the basis of these features, future research should focus on elucidating why the TS and one of its sides are more commonly involved. Among the various anatomical factors that could potentially affect this pathophysiology, we hypothesize that angulation is significant in determining these differences. We also theorize that angulation induces venous stasis in areas surrounding the vascular junctions owing to the sudden alteration of blood flow direction. This could lead to slower blood flow, especially when angulations are extremely acute, thereby potentiating thrombogenesis at such locations. This mechanism is also associated with VSS, which steadily disrupts flow in the pre- and post-stenosis segments.

Furthermore, it is intriguing that certain researchers have investigated the link between angulation and arterial hemodynamics, and endothelial alterations, in coronary and intracranial arteries to elucidate the mechanisms of atherosclerosis development and aneurysm formation, respectively [26, 27–28, 29, 30]. Not long ago, Katakia et al. used 3D-printed branched human coronary arteries, along with computational fluid dynamics analysis and identification of biochemical markers, to explore the influence of coronary artery bifurcation angles on hemodynamic alterations and associated changes in endothelial function [27]. The authors stated that the angular difference in the human coronary artery governs endothelial cell structure and function. Thus, they determined that areas of vessel curvature and branching, depending on the degree of angulation, were susceptible to lower wall shear stress (a paramount regulator of genes beneficial to endothelial function), leading to endothelial cell dysfunction. This was manifested by reduced expression of eNOS, KLF2, and KLF4 (endothelial pro-survival genes), while expression of ICAM1, VCAM1, and P-selectin (inflammatory adhesion molecules) was increased. Even though arteries and veins have obviously distinct characteristics, the insights gained from studies of angulation in arteries should be taken into account in future research endeavors. It is imperative to conduct further investigations to explore the applicability of these methods in studying the intracranial venous circulation, potentially enhancing our understanding of DVSs blood flow-related pathologies.

**Fig. 5:**
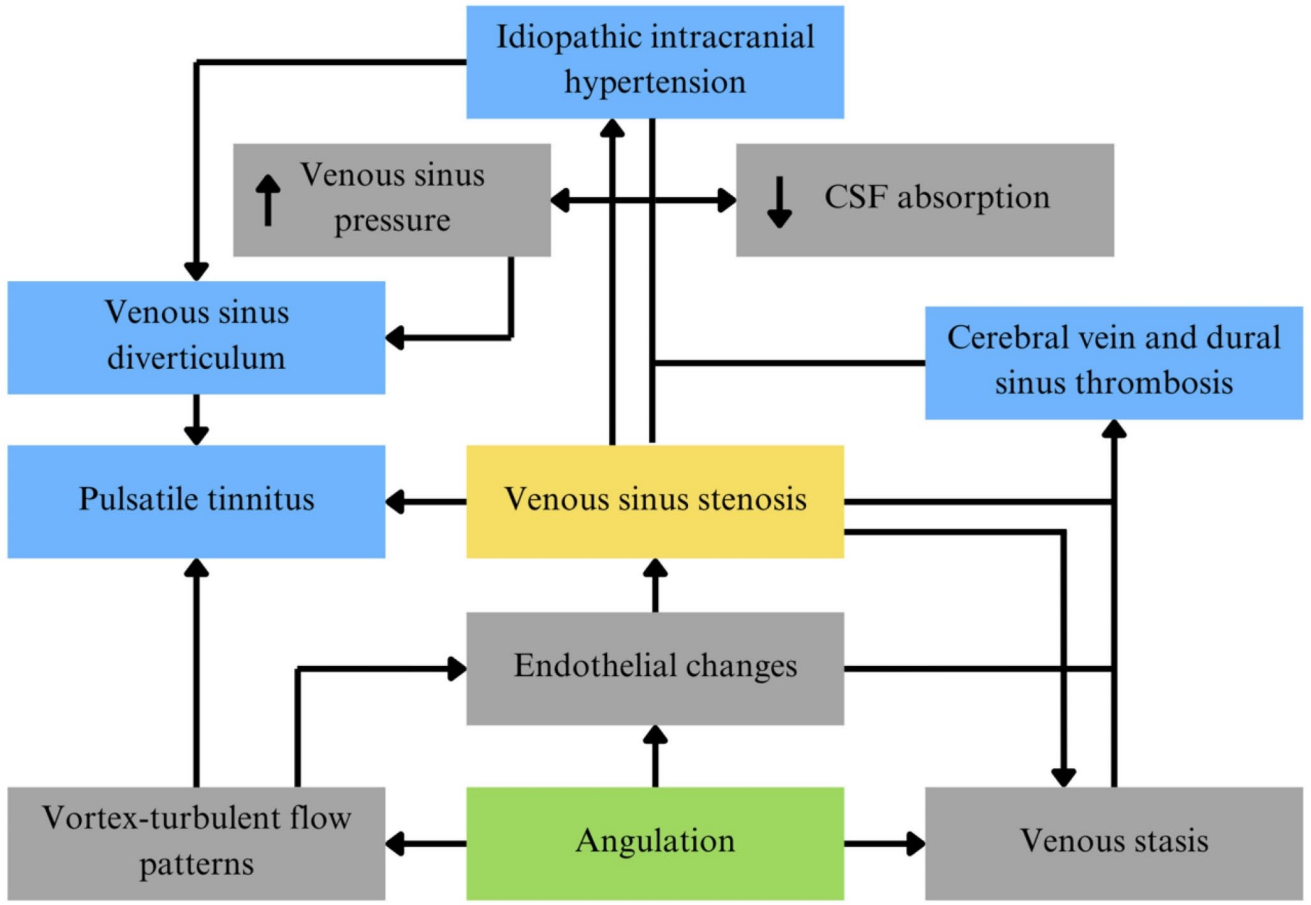
Pathophysiological mechanisms and their association between angulation of the dural venous sinuses and blood flow-related pathologies

VSS is frequently secondary to intrinsic causes such as arachnoid granulations or septa. However, scenarios of unknown etiology have been reported, and the mechanisms discussed have been linked to angulation and its effect on endothelial function. This is likely to contribute to the development of VSS, especially at or near the TS-SSJ [31, 27, 32, 33]. VSS has paramount involvement in the pathophysiological mechanisms of blood flow-related pathologies in DVSs (Fig. 5). It is a highly sensitive radiological indicator in patients with IIH, even being considered as a potential etiology for IIH, particularly among patients who show evidence of VSS and an increased trans-stenotic pressure gradient and have significant resolution of symptoms and normalization of intracranial pressure after venous sinus stenting [34].

IIH is characterized by an opening cerebrospinal fluid (CSF) pressure greater than 25 cm H_2_O on lumbar puncture, coupled with manifestations of elevated intracranial pressure including papilloedema, visual abnormalities, headaches, nausea, vomiting, etc [34]. Notably, CSF composition and neuroimaging typically appear normal, yet the underlying cause remains elusive. The primary pathophysiological mechanisms potentially linked to this entity are CSF hypersecretion, CSF outflow obstruction, reduced CSF absorption, and increased venous sinus pressure [35, 34]. Of these, the latter two are underscored herein because of their indirect connection with angulation through the development of VSS. In addition, these mechanisms are related to VSD and CVST.

VSD is a vascular anomaly frequently observed in patients with VSS and IIH. Increased pressure within the venous sinuses, or secondary factors such as impaired CSF absorption, can eventually give rise to the characteristic outpouching, typically detected on the SS and TS, which is also a significant underlying cause of PT [36, 37]. PT is a type of tinnitus characterized by a subjective perception of auditory sensations, e.g., as “a buzzing, ringing, or whistling” synchronous with the heartbeat [35, 34]. Venous causes encompass the most common vascular etiologies of PT. Moreover, within this group, VSS, IIH, and VSD are often involved in the development of this pathology [35, 34]. Similarly, hemodynamic abnormalities such as vortex-turbulent flow patterns, where angulation is likely to be critical, tend to be related to PT, VSS, and VSD, even leading to the presumption that these abnormal flow patterns are the root of PT [23, 24].

### Limitations

The current study is limited by the absence of clinical history and sociodemographic data from the donors of the heads. The difference in the number of males and females might have influenced the results. Additionally, the angulations were measured using two-dimensional planes. While the current study indicates that angulation constitutes an additional anatomical factor influencing intracranial venous hemodynamics and DVSs blood flow-related pathologies, the precise effect and the potential risk of association between this parameter and the mentioned considerations remain to be fully assessed.

## Conclusions

The DVSs are crucial for maintaining proper venous blood flow within the skull. Understanding the intraluminal behavior of the venous sinus blood and the factors that are likely to affect its normal features could help to elucidate the blood flood-related pathologies of DVSs and potentially determine associations within the pathophysiological mechanisms of such conditions. The results presented in this study show that certain angulations of the DVSs in the posterior cranial fossa can differ significantly between sides and between sexes, but not between types of tissue. Angulation is a key parameter in blood hemodynamics; nevertheless, its influence on the intracranial venous circulation as an intrinsic anatomical factor is yet to be investigated. Future directions should aim to explore the effects of angulation on intracranial venous hemodynamics, and the applicability of our findings to patients with DVS blood flow-related pathologies is also required to extrapolate our results.

## Data Availability

No datasets were generated or analysed during the current study.

## Abbreviations

CSF: Cerebrospinal fluid
CVST: Cerebral vein and dural venous sinus thrombosis
DVS: Dural venous sinus
IIH: Idiopathic intracranial hypertension
PT: Pulsarile tinnitus
SS: Sigmoid sinus
SSS: Superior sagittal sinus
SSS-TSJ: Superior sagital-transverse sinus junction
TS: Transverse sinus
TS-SSJ: Transverse-sigmoid sinus junction
VSD: Venous sinus diverticulum
VSS: Venous sinus stenosis

## References

[1] GBD 2019 Stroke Collaborators (2021) Global, regional, and national burden of stroke and its risk factors, 1990–2019: a systematic analysis for the global burden of Disease Study 2019. Lancet Neurol 20:795–820. 10.1016/S1474-4422(21)00252-0 34487721 10.1016/S1474-4422(21)00252-0 PMC8443449

[2] de Simone G, Stranges S, Gentile I (2021) Incidence of cerebral venous thrombosis and COVID-19 vaccination: possible causal effect or just chance? Eur Heart J Cardiovasc Pharmacother 7:e77–e78. 10.1093/ehjcvp/pvab036 33930114 10.1093/ehjcvp/pvab036 PMC8135678

[3] Liu J, Cao F, Luo C, Guo Y, Yan J (2023) Stroke following Coronavirus Disease 2019 Vaccination: evidence based on different designs of real-World studies. J Infect Dis 228:1336–1346. 10.1093/infdis/jiad306 37536364 10.1093/infdis/jiad306

[4] Bezerra GMS, Cavalcante YDS, Matos-Neto PR et al (2022) Cerebral venous thrombosis in Latin America: a critical review of risk factors, clinical and radiological characteristics. Front Neurol 13:1017565. 10.3389/fneur.2022.1017565 36388216 10.3389/fneur.2022.1017565 PMC9649826

[5] Dentali F, Poli D, Scoditti U et al (2012) Long-term outcomes of patients with cerebral vein thrombosis: a multicenter study. J Thromb Haemost 10:1297–1302. 10.1111/j.1538-7836.2012.04774.x 22578023 10.1111/j.1538-7836.2012.04774.x

[6] Duman T, Uluduz D, Midi I et al (2017) A Multicenter Study of 1144 patients with cerebral venous thrombosis: the VENOST Study. J Stroke Cerebrovasc Dis 26:1848–1857. 10.1016/j.jstrokecerebrovasdis.2017.04.020 28583818 10.1016/j.jstrokecerebrovasdis.2017.04.020

[7] Ferro JM, Canhão P, Stam J, Bousser MG, Barinagarrementeria F (2004) ISCVT investigators. Prognosis of cerebral vein and dural sinus thrombosis: results of the International Study on Cerebral Vein and Dural Sinus thrombosis (ISCVT). Stroke 35:664–670. 10.1161/01.STR.0000117571.76197.26 14976332 10.1161/01.STR.0000117571.76197.26

[8] Ferro JM, Lopes MG, Rosas MJ, Ferro MA, Fontes J (2002) Cerebral venous thrombosis portugese Collaborative Study Group. Long-term prognosis of cerebral vein and dural sinus thrombosis. Results of the VENOPORT study. Cerebrovasc Dis 13:272–278. 10.1159/000057855 12011553 10.1159/000057855

[9] Jianu DC, Jianu SN, Munteanu G, Dan FT, Bârsan C (2018) Cerebral vein and Dural Sinus thrombosis. Ischemic stroke of Brain. Intech Open, London, UK. Chap. 3

[10] Paybast S, Mohamadian R, Emami A et al (2022) Safety and efficacy of endovascular thrombolysis in patients with acute cerebral venous sinus thrombosis: a systematic review. Interv Neuroradiol. 10.1177/15910199221143418. Published online December 5, 2022 36471504 10.1177/15910199221143418 PMC11569481

[11] Altafulla JJ, Prickett J, Iwanaga J, Dumont AS, Tubbs RS (2020) Intraluminal anatomy of the transverse sinus: implications for endovascular therapy. Anat Cell Biol 53:393–397. 10.5115/acb.20.041 32647072 10.5115/acb.20.041 PMC7769094

[12] Iwanaga J, Courville E, Anand MK et al (2020) Chordae Willisii within the transverse sinus: morphologic study. World Neurosurg 139:e38–e44. 10.1016/j.wneu.2020.03.024 32173547 10.1016/j.wneu.2020.03.024

[13] Strydom MA, Briers N, Bosman MC, Steyn S (2010) The anatomical basis of venographic filling defects of the transverse sinus. Clin Anat 23:153–159. 10.1002/ca.20911 20014389 10.1002/ca.20911

[14] Cardona JJ, Iwanaga J, Chaiyamoon A et al (2024) Angulation of the dural venous sinuses of the posterior cranial fossa: anatomical study with clinical and surgical applications. Clin Anat 37:546–554. 10.1002/ca.24154 38475991 10.1002/ca.24154

[15] Iwanaga J, Singh V, Takeda S et al (2022) Standardized statement for the ethical use of human cadaveric tissues in anatomy research papers: recommendations from Anatomical Journal editors-in-Chief. Clin Anat 35:526–528. 10.1002/ca.23849 35218594 10.1002/ca.23849

[16] Okudera T, Huang YP, Ohta T et al (1994) Development of posterior fossa dural sinuses, emissary veins, and jugular bulb: morphological and radiologic study. AJNR Am J Neuroradiol 15:1871–1883 7863937 PMC8334261

[17] Yamaguchi K, Goto N, Nara T (1998) [Development of the human fetus cerebellum: a volumetric study of the cerebellar structures]. No Hattatsu 20:3–9 3348919

[18] Hirakoh G (1962) On the fossa jugularis and outflow cranial venous blood through it. J Kurume Med Ass 25:965–971

[19] Kaplan HA, Browder J (1976) Neurosurgical consideration of some features of the cerebral dural sinuses and their tributaries. Clin Neurosurg 23:155–169. 10.1093/neurosurgery/23.cn_suppl_1.155 975676 10.1093/neurosurgery/23.cn_suppl_1.155

[20] Schuchardt F, Schroeder L, Anastasopoulos C et al (2015) In vivo analysis of physiological 3D blood flow of cerebral veins. Eur Radiol 25:2371–2380. 10.1007/s00330-014-3587-x 25638218 10.1007/s00330-014-3587-x

[21] Mehta NR, Jones L, Kraut MA, Melhem ER (2000) Physiologic variations in dural venous sinus flow on phase-contrast MR imaging. AJR Am J Roentgenol 175:221–225. 10.2214/ajr.175.1.1750221 10882276 10.2214/ajr.175.1.1750221

[22] Dai C, Zhao P, Ding, H et al (2024) Cerebral sinus hemodynamics in adults revealed by 4D Flow MRI. J Magn Reson Imaging. 10.1002/jmri.29210. Published online January 18, 2024 38235948 10.1002/jmri.29210

[23] Amans MR, Haraldsson H, Kao E et al (2018) MR venous Flow in Sigmoid Sinus Diverticulum. AJNR Am J Neuroradiol 39:2108–2113. 10.3174/ajnr.A5833 30309843 10.3174/ajnr.A5833 PMC6239885

[24] Hong Z, Liu X, Ding H et al (2022) Flow patterns in the venous sinus of pulsatile tinnitus patients with transverse sinus stenosis and underlying vortical flow as a causative factor. Comput Methods Programs Biomed 227:107203. 10.1016/j.cmpb.2022.107203 36370596 10.1016/j.cmpb.2022.107203

[25] Li Y, Chen H, He L et al (2018) Hemodynamic assessments of venous pulsatile tinnitus using 4D-flow MRI. Neurology 91:e586–e593. 10.1212/WNL.0000000000005948 29997192 10.1212/WNL.0000000000005948

[26] Beier S, Ormiston J, Webster M et al (2016) Impact of bifurcation angle and other anatomical characteristics on blood flow - A computational study of non-stented and stented coronary arteries. J Biomech 49:1570–1582. 10.1016/j.jbiomech.2016.03.038 27062590 10.1016/j.jbiomech.2016.03.038

[27] Katakia YT, Kanduri S, Bhattacharyya R et al (2022) Angular difference in human coronary artery governs endothelial cell structure and function. Commun Biol 5:1044. 10.1038/s42003-022-04014-3 36183045 10.1038/s42003-022-04014-3 PMC9526720

[28] Lauric A, Hippelheuser JE, Malek AM (2018) Induction of aneurysmogenic high positive wall shear stress gradient by wide angle at cerebral bifurcations, independent of flow rate. J Neurosurg 131:442–452. 10.3171/2018.3.JNS173128 30095336 10.3171/2018.3.JNS173128

[29] Malek AM, Hippelheuser JE, Lauric A (2021) Vortex formation and associated aneurysmogenic transverse rotational shear stress near the apex of wide-angle cerebral bifurcations. J Neurosurg 136:1726–1737. 10.3171/2021.6.JNS204385 34715656 10.3171/2021.6.JNS204385

[30] Wong KKL, Wu J, Liu G, Huang W, Ghista DN (2020) Coronary arteries hemodynamics: effect of arterial geometry on hemodynamic parameters causing atherosclerosis. Med Biol Eng Comput 58:1831–1843. 10.1007/s11517-020-02185-x 32519006 10.1007/s11517-020-02185-x

[31] Cardona JJ, Iwanaga J, Heiferman DM et al (2023) Dural sinus septum as an underlying cause of intrinsic venous sinus stenosis: Anatomical, clinical, and stent placement considerations. Interv Neuroradiol Published online October 6, 2023. 10.1177/1591019923120604010.1177/1591019923120604037801551

[32] Raynald, Chen N, Yang H et al (2024) Intravascular ultrasound characteristics of different types of stenosis in idiopathic intracranial hypertension with venous sinus stenosis. J Neurointerv Surg 16:506–511. 10.1136/jnis-2023-020345 37355254 10.1136/jnis-2023-020345

[33] Zhao Y, Zhang X, Lv B et al (2024) Dural sinus septum: an underlying cause of cerebral venous sinus stenting failure and complications. Stroke Vasc Neurol 9:174–180. 10.1136/svn-2023-002407 37433695 10.1136/svn-2023-002407 PMC11103156

[34] Pandey A, Schreiber C, Garton ALA et al (2024) Foundations of the diagnosis and management of idiopathic intracranial hypertension and pulsatile Tinnitus. World Neurosurg 184:361–371. 10.1016/j.wneu.2023.12.125 38590070 10.1016/j.wneu.2023.12.125

[35] Gurney SP, Ramalingam S, Thomas A, Sinclair AJ, Mollan SP (2020) Exploring the current management idiopathic intracranial hypertension, and understanding the role of dural venous sinus stenting. Eye Brain 12:1–13. 10.2147/EB.S193027 32021528 10.2147/EB.S193027 PMC6969694

[36] Cummins DD, Caton MT, Hemphill K et al (2023) Cerebrovascular pulsatile tinnitus: causes, treatments, and outcomes in 164 patients with neuroangiographic correlation. J Neurointerv Surg 15:1014–1020. 10.1136/jnis-2022-019259 36190940 10.1136/jnis-2022-019259

[37] Narsinh KH, Hui F, Duvvuri M, Meisel K, Amans MR (2022) Management of vascular causes of pulsatile tinnitus. J Neurointerv Surg 14:1151–1157. 10.1136/neurintsurg-2021-018015 35145036 10.1136/neurintsurg-2021-018015 PMC9363535

[38] Iwanaga J, Singh V, Ohtsuka A et al (2021) Acknowledging the use of human cadaveric tissues in research papers: recommendations from anatomical journal editors. Clin Anat 34:2–4. 10.1002/ca.23671 32808702 10.1002/ca.23671

